# Three distinct trajectories of red blood cell distribution width and their significant associations with mortality in sepsis patients: a group-based trajectory modeling study with validation

**DOI:** 10.3389/fmed.2026.1816360

**Published:** 2026-04-13

**Authors:** Lei Cai, Yuwei Hua, Wei Lu, Haozhen Bing, Qinfen Gao, Wei Zhang

**Affiliations:** 1 Kunming Medical University, Kunming, China; 2 The First Affiliated Hospital of Kunming Medical University, Kunming, China; 3 First People's Hospital of Yunnan, Kunming, China

**Keywords:** group-based trajectory modeling, prognosis, red cell distribution width, sepsis, trajectory

## Abstract

**Background:**

The red cell distribution width (RDW) is a recognized prognostic marker in sepsis, yet its dynamic changes over time and their relationship with outcomes remain unexplored. This study aimed to identify distinct RDW trajectories during the early phase of sepsis and evaluate their association with mortality.

**Methods:**

We conducted a retrospective cohort study using data from the MIMIC-IV database (*n* = 3,813) as the derivation cohort and from the First Affiliated Hospital of Kunming Medical University (*n* = 467) for external single-center validation. Sepsis patients with at least seven RDW measurements within the first 10 days of hospitalization were included. Group-based trajectory modeling (GBTM) was employed to identify RDW trajectories.

**Results:**

A three-trajectory model was selected based on model fit indices and clinical interpretability: Trajectory 1 (Slow-Decrease, 32.97%), Trajectory 2 (Slow-Increase, 43.30%), and Trajectory 3 (Fluctuating-Rapid Decrease, 23.73%). In our study, Cox models adjusted for confounders revealed that, compared to Trajectory 1, Trajectory 3 was independently associated with significantly increased 30-day (HR 1.47, 95% CI 1.17–1.84) and 90-day mortality (HR 1.54, 95% CI 1.25–1.88). Conversely, Trajectory 2 was associated with the most favorable survival rates. Kaplan–Meier analysis consistently showed the highest mortality in the Trajectory 3 group. External validation confirmed the model’s robustness and the consistent prognostic value of the identified trajectories.

**Conclusion:**

This study is the first to apply trajectory modeling to identify three dynamic RDW trajectories with significant prognostic stratification in sepsis patients. Among them, the “fluctuating-rapid decline” trajectory is an independent risk factor for both 30-day and 90-day mortality. However, due to the limitation of RDW testing frequency, the study may represent a group with more severe illness, which may limit the generalizability of the conclusions. This discovery elevates the conventional indicator RDW into a dynamic and practical bedside risk stratification tool, which may assist clinicians in early identification of high-risk patients.

## 1 Introduction

Sepsis is a life-threatening organ dysfunction caused by a dysregulated host response to infection. It poses a major threat to human life and safety. Among the 48.9 million sepsis cases reported globally in 2017, the mortality rate was approximately 22.5%, accounting for nearly 20% of global deaths that year (1). Concurrently, sepsis is one of the leading causes of in-hospital mortality, with a fatality rate of 18.7% among hospitalized sepsis patients, a figure that can reach up to 55.7% in the Intensive Care Unit (ICU) (2). Despite established frameworks like Sepsis-3 and the Surviving Sepsis Campaign, accurately predicting outcomes for sepsis patients remains challenging.

The Red Blood Cell Distribution Width (RDW) is a common parameter in a complete blood count, indicating the variability in the size of circulating red blood cells. Historically, RDW was primarily used for the diagnosis and differential diagnosis of iron-deficiency anemia and other chronic anemias. Recent studies, however, have found that elevated RDW is associated with increased mortality in various conditions, including septic cardiomyopathy (3), acute myocardial infarction (4, 5), heart failure (6, 7), cardiac arrest (8), and acute pulmonary embolism (9). Nevertheless, previous research has predominantly analyzed RDW at single, fixed time points, overlooking the relationship between its dynamic changes and patient prognosis in sepsis. Furthermore, as a readily available component of the routine complete blood count used clinically, RDW facilitates easy serial monitoring. This study aims to identify distinct RDW trajectories in sepsis patients using Group-Based Trajectory Modeling (GBTM), a method that accommodates unbalanced panels and missing data.

## 2 Methods

### 2.1 Data sources

Patient data for the modeling cohort were extracted from the MIMIC-IV database, version 3.1 (application number: 66772901). The validation cohort consists of inpatients admitted to the First Affiliated Hospital of Kunming Medical University (KMUFAH) from 2018 to 2025. The MIMIC-IV database is a publicly available electronic health database containing clinical data from approximately 300,000 patients admitted to the Beth Israel Deaconess Medical Center (BIDMC) in Boston between 2008 and 2019. The database has been approved by the Institutional Review Board (IRB) of the Massachusetts Institute of Technology (MIT). All protected health information has been de-identified; specific records (e.g., dates of birth, admission, and discharge) were encrypted to protect patient privacy, thus waiver of informed consent. The data from the First Affiliated Hospital of Kunming Medical University has been approved by the Hospital Ethics Committee. This retrospective study was conducted in accordance with the Declaration of Helsinki. Due to the retrospective nature of the study and the use of anonymized data (for MIMIC-IV) and data collected from routine clinical care with waived consent approved by the ethical committee (for KMUFAH data), the requirement for informed consent was waived by the respective IRBs/Ethics Committees (MIT IRB and the Ethics Committee of the First Affiliated Hospital of Kunming Medical University).”

### 2.2 Study population

**Figure 1:**
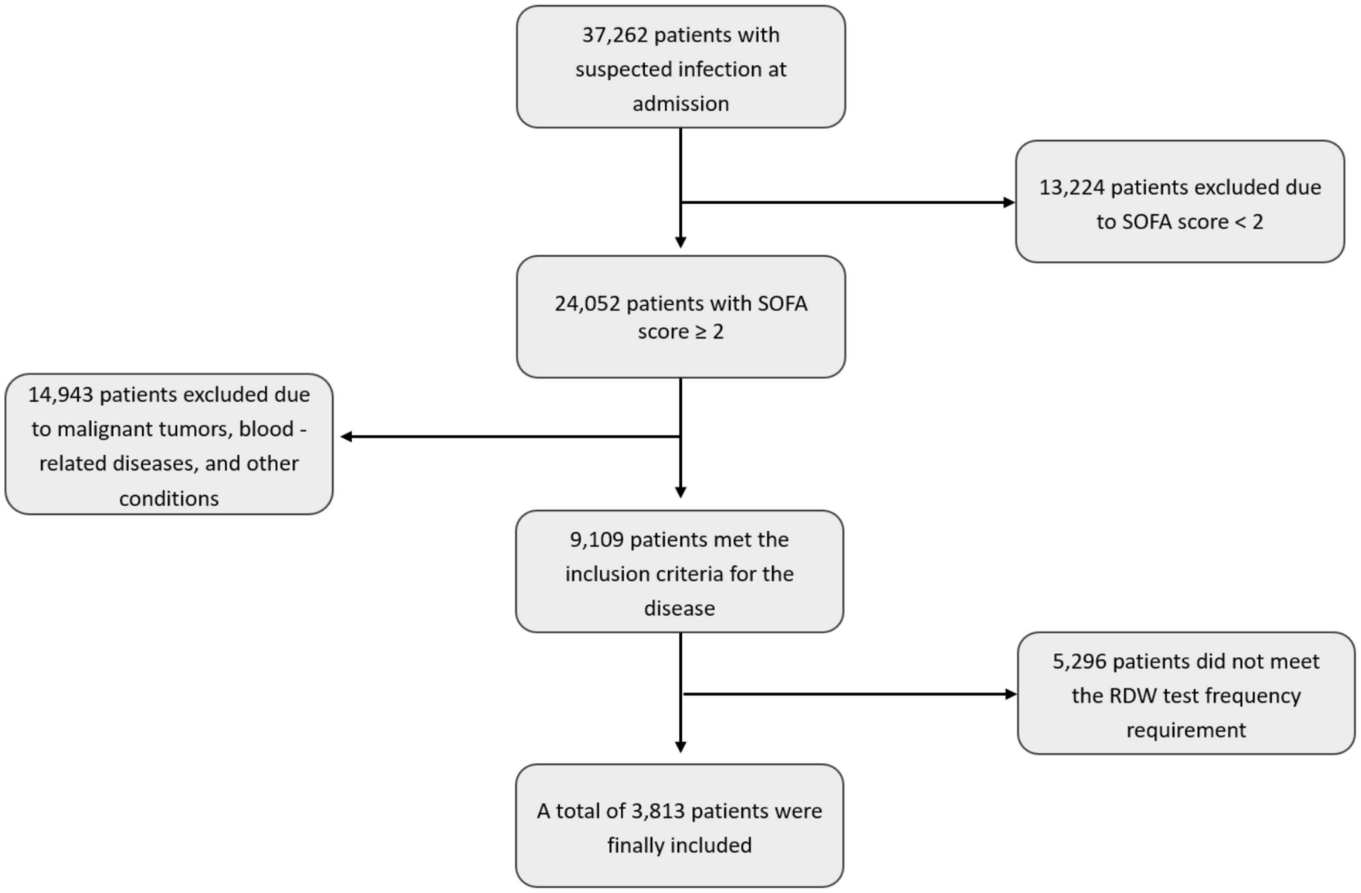
Flowchart of patient selection from the MIMIC-IV database.

The inclusion criteria were as follows: (i) meeting the Sepsis-3.0 diagnostic criteria [confirmed or suspected infection plus an increase in Sequential Organ Failure Assessment (SOFA) score ≥2]; (ii) age ≥18 years; and (iii) having at least seven RDW measurements within the first 10 days of hospitalization. The exclusion criteria were as follows: (i) a history of comorbidities including malignant tumors, hematological diseases, chronic obstructive pulmonary disease (COPD), obstructive sleep apnea, acute or chronic blood loss, or blood transfusion; and (ii) excessive missing data. The detailed patient screening process is shown in Figure 1.

### 2.3 Clinical data

The study extracted the following information from the MIMIC-IV database for eligible patients for statistical analysis: basic demographic data, diagnoses, outcomes, survival time, RDW values and their corresponding measurement times, ICU length of stay, Sequential Organ Failure Assessment (SOFA) score, Glasgow Coma Scale (GCS) score, Acute Physiology Score III (APS III), Oxford Acute Severity of Illness Score (OASIS), Model for End-Stage Liver Disease (MELD) score, and Charlson Comorbidity Index (CCI).

### 2.4 Statistical analysis

Group-based trajectory modeling (GBTM) was employed to identify distinct RDW trajectories. The optimal number of trajectory groups was selected based on the smallest Akaike Information Criterion (AIC) and Bayesian Information Criterion (BIC), combined with clinical interpretability. Given the minimal amount of missing data, median imputation was used. Each patient was assigned to a trajectory group based on the model’s posterior probability. The Kolmogorov–Smirnov test was used to assess the normality of data distribution. Normally distributed continuous data are presented as mean ± standard deviation and were compared using one-way analysis of variance (ANOVA). Non-normally distributed continuous data are presented as median (interquartile range) and were compared using the Kruskal–Wallis *H*-test. The Kaplan–Meier method was used to plot survival curves, and Cox proportional hazards models were applied to analyze the impact of different covariates on survival. All statistical analyses were performed using R software (version 4.4.2).

## 3 Results

### 3.1 Group-based trajectory modeling

**Figure 2:**
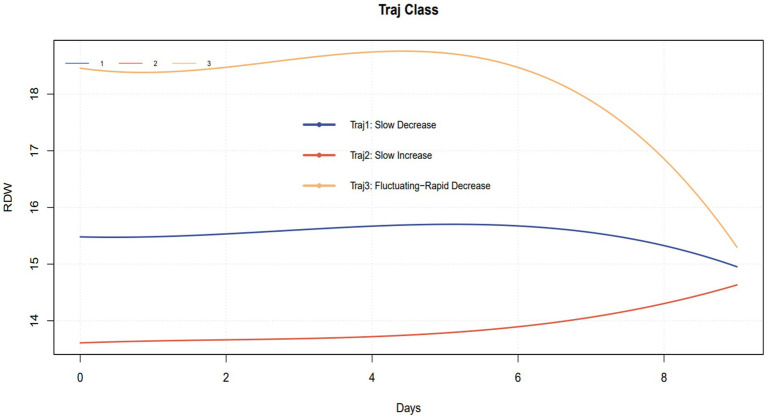
Identified RDW trajectories in sepsis patients.

We employed group-based trajectory modeling (GBTM) to identify distinct RDW trajectories. Based on the minimization criteria for Akaike Information Criterion (AIC) and Bayesian Information Criterion (BIC), along with clinical interpretability, a three-trajectory model was selected (Figure 2). Trajectory 1 (Traj 1): Slow-Decrease group, included 1,257 patients (32.97%), characterized by a gradual decrease in RDW. Trajectory 2 (Traj 2): Slow-Increase group, included 1,651 patients (43.30%), characterized by a gradual increase in RDW. Trajectory 3 (Traj 3): Fluctuating-Rapid Decrease group, included 905 patients (23.73%), characterized by a baseline RDW value above normal levels, which fluctuated initially followed by a rapid decrease.

### 3.2 Baseline characteristics of the patients

Patients were assigned to the Traj 1 (*n* = 1,257), Traj 2 (*n* = 1,651), and Traj 3 (*n* = 905) groups based on the maximum posterior probability. The Kruskal–Wallis *H*-test indicated significant differences in baseline characteristics among the different trajectory groups. Patients in the Traj 3 group had the highest SOFA, APS III, and MELD scores, alongside the highest in-hospital, 30-day, and 90-day mortality rates. Regarding ICU stay, the Traj 3 group had the shortest length of stay. Conversely, the Traj 2 group had the longest hospital and ICU lengths of stay, and the lowest APS III scores, in-hospital mortality, 30-day mortality, and 90-day mortality rates (Table 1).

**Table 1:** Clinical characteristics of patients with different RDW trajectories.

Variables	Traj 1 (*n* = 1,257)	Traj 2 (*n* = 1,651)	Traj 3 (*n* = 905)	*p*
Age (years)	66.0 (54.0, 77.0)	66.0 (53.0, 77.0)	65.0 (55.0, 76.0)	*p* = 0.466
Sex (male)	641 (51.0%)	886 (53.7%)	448 (49.5%)	*p* = 0.103
Hospital LOS (days)	14.5 (9.9, 23.3)	15.4 (10.1, 25.4)	14.9 (10.4, 25.9)	*p* = 0.028
ICU LOS (days)	4.7 (2.3, 10.0)	5.8 (2.8, 11.9)	4.0 (2.0, 9.2)	*p* < 0.001
SOFA	3.0 (2.0, 4.0)	3.0 (2.0, 4.0)	4.0 (2.0, 5.0)	*p* < 0.001
GCS	15.0 (14.0, 15.0)	15.0 (14.0, 15.0)	15.0 (14.0, 15.0)	*p* = 0.009
APSIII	52.0 (41.0, 66.0)	46.0 (34.0, 58.0)	57.0 (46.0, 71.0)	*p* < 0.001
OASIS	35.0 (29.0, 41.0)	34.0 (28.0, 39.0)	35.0 (29.0, 41.0)	*p* < 0.001
MELD	15.0 (10.0, 21.0)	11.0 (8.0, 16.0)	20.0 (12.0, 25.0)	*p* < 0.001
SIRS	3.0 (2.0, 3.0)	3.0 (2.0, 3.0)	3.0 (2.0, 3.0)	*p* = 0.110
Charlson Comorbidity Index	6.0 (4.0, 8.0)	5.0 (3.0, 7.0)	6.0 (5.0, 8.0)	*p* < 0.001
In-hospital mortality	183 (14.6%)	181 (11.0%)	217 (24.0%)	*p* = 0.121
30-day mortality	149 (11.9%)	147 (8.9%)	166 (18.3%)	*p* < 0.001
90-day mortality	178 (14.2%)	180 (10.9%)	209 (23.1%)	*p* < 0.001

Data are presented as median (interquartile range) or *n* (%). LOS, length of stay; SOFA, Sequential Organ Failure Assessment; GCS, Glasgow Coma Scale; APS III, Acute Physiology Score III; OASIS, Oxford Acute Severity of Illness Score; MELD, Model for End-Stage Liver Disease; SIRS, systemic inflammatory response syndrome.

### 3.3 Cox proportional hazards models and Kaplan–Meier survival curves

Cox proportional hazards models and Kaplan–Meier survival curves were used to analyze the association between different RDW trajectories and mortality. In the unadjusted model, compared to the Traj 1 group, the Traj 3 group was significantly associated with increased 30-day (HR 1.60, 95% CI 1.29–2.00, *p* < 0.001) and 90-day mortality (HR 1.71, 95% CI 1.40–2.09, *p* < 0.001). Conversely, the Traj 2 group was significantly associated with decreased 30-day (HR 0.74, 95% CI 0.59–0.93, *p* = 0.010) and 90-day mortality (HR 0.76, 95% CI 0.62–0.93, *p* = 0.008). Even after adjusting for confounding factors, the Traj 3 group remained significantly associated with increased 30-day and 90-day mortality, the Traj 2 group remained significantly associated with decreased 30-day and 90-day mortality, while this protective effect disappeared after multivariable adjustment, This may be because scores that directly quantify organ dysfunction and disease severity, such as SOFA and APS III, accounted for the core prognostic information, thereby diminishing the significance of Traj 2 and resulting in the loss of its independent effect. Consistent with the Cox model results, the Kaplan–Meier survival curves demonstrated that the Traj 3 group had the highest mortality rates at both 30 and 90 days, whereas the Traj 2 group had the lowest mortality rates at both time points.

**Figure 3:**
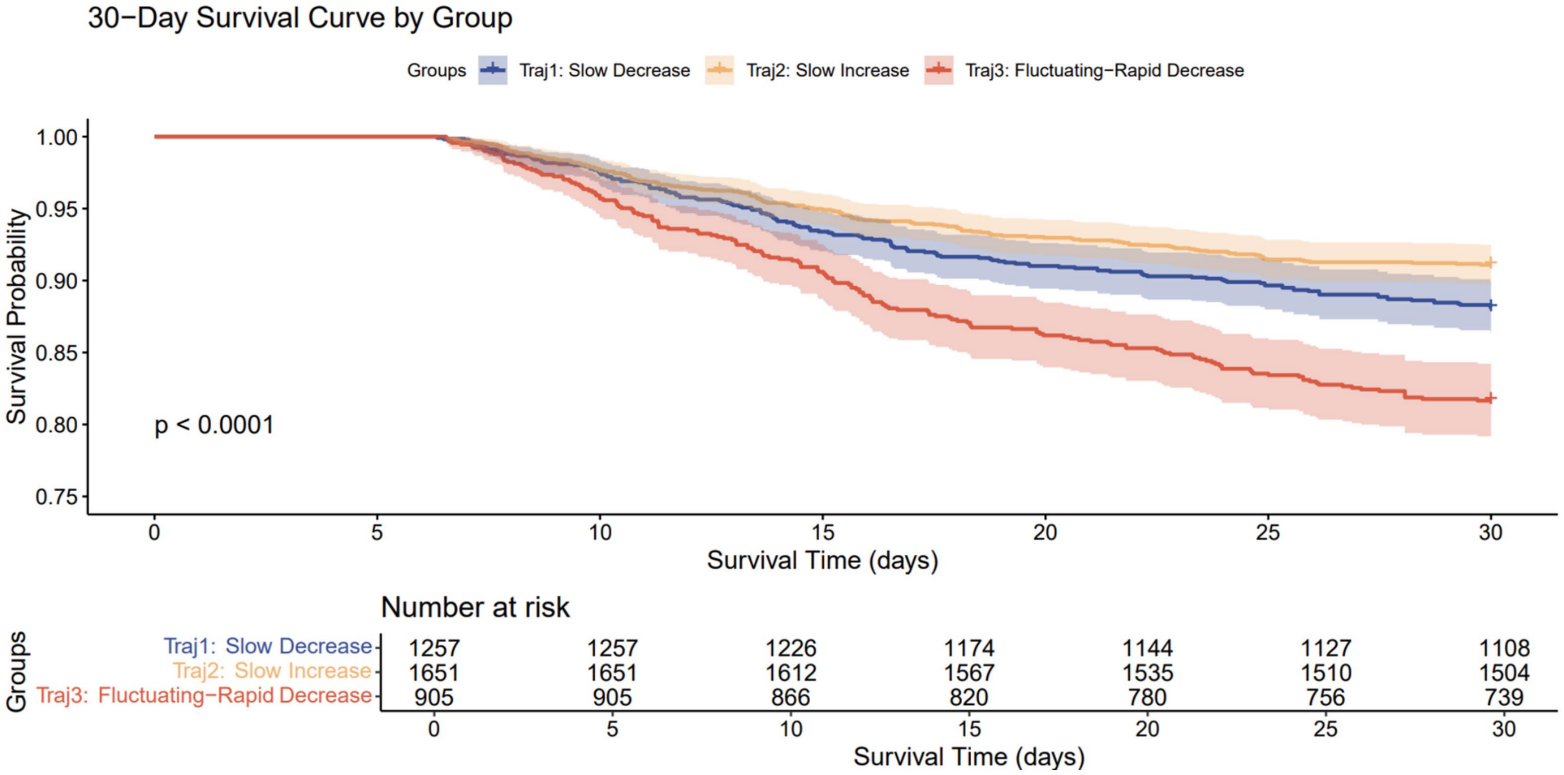
30-day Kaplan–Meier survival curves for patients with different trajectories.

**Figure 4:**
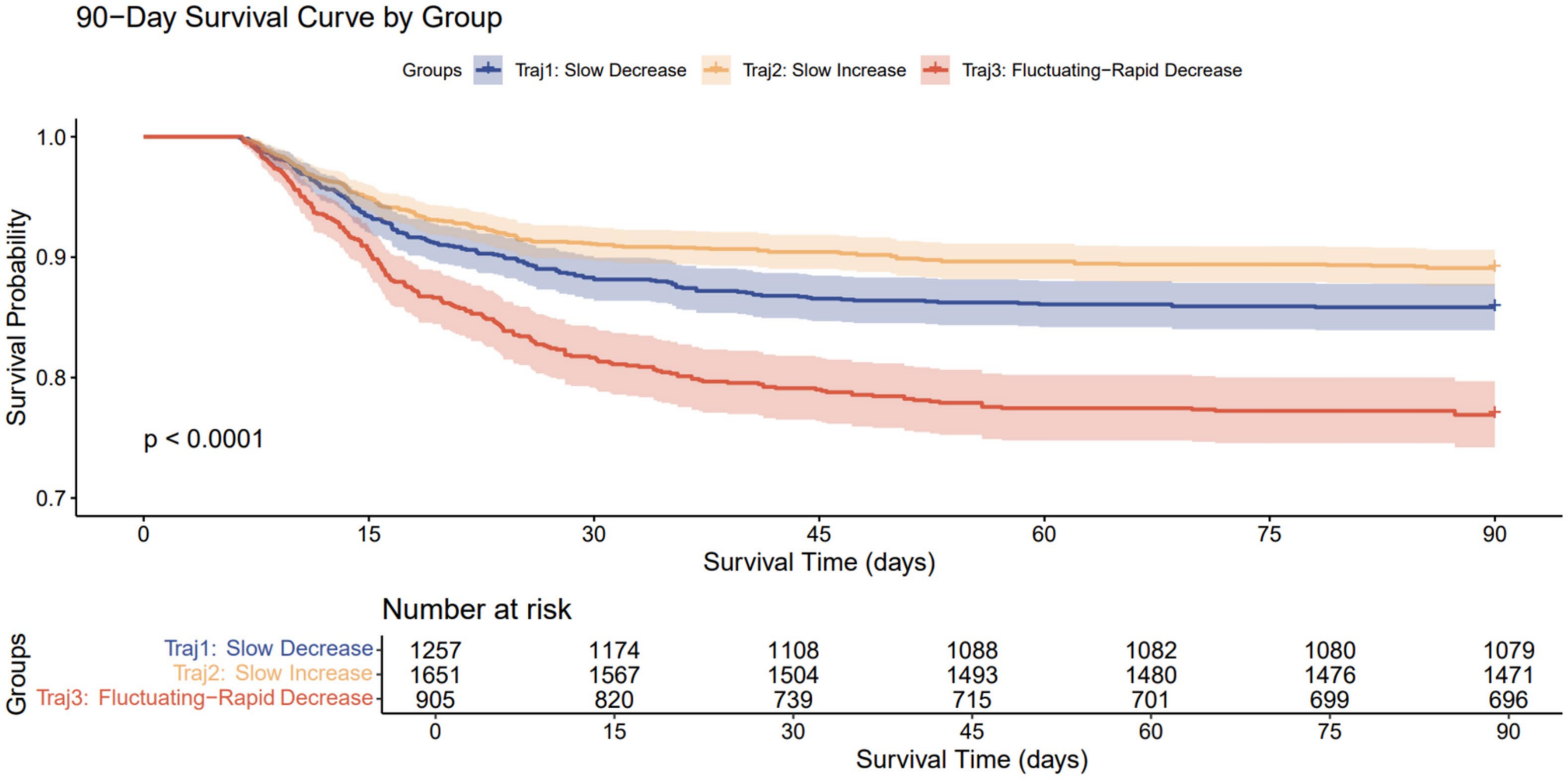
90-day Kaplan–Meier survival curves for patients with different trajectories.

In the Cox proportional hazards models, we assessed the proportional hazards assumption using the Schoenfeld residual test. The results showed that the RDW trajectory satisfied this assumption in all models (all *p* > 0.05, see Supplementary Tables S1, S2 for details), indicating that the strength of its association with the risk of death remained stable throughout the observation period (Table 2; Figures 3, 4).

**Table 2:** Univariable and multivariable Cox proportional hazards models for different RDW trajectories.

	Traj	Crude	Model I	Model II	Model III
30-day mortality	Traj 1	Ref	Ref	Ref	Ref
	Traj 2	0.74 (0.59, 0.93, *p =* 0.010)	0.74 (0.59, 0.93, *p =* 0.009)	0.77 (0.61, 0.97, *p* = 0.027)	0.93 (0.74, 1.18, *p* = 0.560)
	Traj 3	1.60 (1.29, 2.00, *p*<0.001)	1.65 (1.33, 2.06, *P*<0.001)	1.58 (1.27, 1.98, *P*<0.001)	1.47 (1.17, 1.84, *P*<0.001)
90-day mortality	Traj 1	Ref	Ref	Ref	Ref
	Traj 2	0.76 (0.62, 0.93, *P* = 0.008)	0.74 (0.60, 0.91, *p* = 0.005)	0.78 (0.63, 0.96, *P* = 0.017)	0.94 (0.76, 1.16, *p* = 0.543)
	Traj 3	1.71 (1.40, 2.09, *P*<0.001)	1.75 (1.43 2.13, *P*<0.001)	1.67 (1.36, 2.04, *P*<0.001)	1.54 (1.25 1.88, *P*<0.001)

Crude: unadjusted model. Model I: adjusted for age, sex, and ICU length of stay. Model II: adjusted for variables in Model I plus SOFA score, GCS score, and SIRS score. Model III: adjusted for variables in Model II plus APS III score, OASIS score, and Charlson Comorbidity Index.

### 3.4 C-index, likelihood ratio (LR) test, and net reclassification improvement (NRI)

We also systematically evaluated the incremental predictive value of incorporating RDW dynamic trajectory groups into different clinical prognostic models by using the C-index, LR test, and NRI.

First, we used the C-index and likelihood ratio test to assess the improvement in model discrimination (C-index) and model fit brought by the RDW trajectory groups. After introducing the RDW trajectory groups into the three progressively adjusted baseline models (Table 3), their C-index values all achieved statistically significant improvements (all *p* <0.05). In the most basic Model I, adding RDW trajectories increased the C-index by 3.6%. In Model II, which included SOFA and SIRS scores, adding RDW trajectories increased the C-index by 1.8%. Even in the most comprehensive Model III, the C-index still significantly increased by 0.65%. The likelihood ratio test (Table 4) provided stronger evidence, indicating that incorporating trajectory groups into all three models extremely significantly improved the overall goodness-of-fit of the models (all *p* < 0.001). The improvement in C-index combined with the significance of the likelihood ratio test jointly confirmed that RDW trajectory grouping is a predictive marker with significant incremental value, independent of traditional prognostic factors.

**Table 3:** Improvement in model discrimination (C-index) by incorporating RDW trajectory groups.

Comparison model	C-index of model	C-index of Model + Traj group	C-index (95% CI)	*p*
Model I vs Model I + Traj group	0.627	0.663	0.0355 (0.0184, 0.0527)	<0.001
Model II vs Model II + Traj group	0.664	0.683	0.0183 (0.0051, 0.0311)	0.004
Model III vs Model III + Traj Group	0.716	0.723	0.0065 (0.0007, 0.0125)	0.028

Model I: adjusted for age, sex, and ICU length of stay. Model II: adjusted for variables in Model I plus SOFA score, GCS score, and SIRS score. Model III: adjusted for variables in Model II plus APS III score, OASIS score, and Charlson Comorbidity Index. C-index, concordance index; CI, confidence interval, calculated via the Bootstrap method (1,000 resamples). All comparisons are referenced to the base model. The *p*-value is used to test the statistical significance of the C-index difference.

**Table 4:** Likelihood ratio test results for incorporating RDW trajectory groups.

Comparison model	*χ* ^2^	*p*
Model I VS Model I + Traj group	78.75	<0.001
Model II VS Model II + Traj group	60.66	<0.001
Model III VS Model III + Traj group	28.13	<0.001

Model I: adjusted for age, sex, and ICU length of stay. Model II: adjusted for variables in Model I plus SOFA score, GCS score, and SIRS score. Model III: adjusted for variables in Model II plus APS III score, OASIS score, and Charlson Comorbidity Index.

Then, we used the Net Reclassification Improvement (NRI) to further elucidate the specific pattern of how trajectory groups improved predictive ability, with the results varying as model complexity increased: In Model I and Model II, incorporating the trajectory groups brought about significant overall net reclassification improvement (overall NRI 7.47 and 5.79%, respectively, with the lower limit of 95% CI > 0). These improvements were almost entirely contributed by the non-event NRI-, indicating that the new model can help existing scores and models more accurately distinguish low-risk patients. In Model III, the overall NRI was a slight negative value (−1.57, 95% CI does not include 0). This suggests that when the baseline model already incorporates comprehensive static clinical assessments (e.g., SOFA, APS III), the net benefit of adding the dynamic information from RDW trajectories for reclassifying patients’ risk becomes limited. This may be because part of the physiological processes (e.g., inflammation) reflected by RDW trajectories are already captured to some extent by scores such as SOFA and APS III (Table 5).

**Table 5:** Net reclassification improvement (NRI) analysis after incorporating RDW trajectory groups (based on 30-day mortality).

Comparison model	Overall NRI (95% CI)	Event NRI+ (95% CI)	Non-event NRI− (95% CI)
Model I vs Model I + Traj group	0.0747 (0.0550, 0.0938)	−0.0052 (−0.0205, 0.0103)	0.0799 (0.0687, 0.0911)
Model II vs Model II + Traj group	0.0579 (0.0352, 0.0786)	−0.0121 (−0.0304, 0.0052)	0.0699 (0.0573, 0.0821)
Model III vs Model III + Traj group	−0.0157 (−0.0286, −0.0031)	0.0069 (−0.0017, 0.0170)	−0.0226 (−0.0313, −0.0145)

Model I: adjusted for age, sex, and ICU length of stay. Model II: adjusted for variables in Model I plus SOFA score, GCS score, and SIRS score. Model III: adjusted for variables in Model II plus APS III score, OASIS score, and Charlson Comorbidity Index. NRI, net reclassification improvement. Overall NRI = event NRI+ + non-event NRI−.

### 3.5 External single-center validation

**Figure 5:**
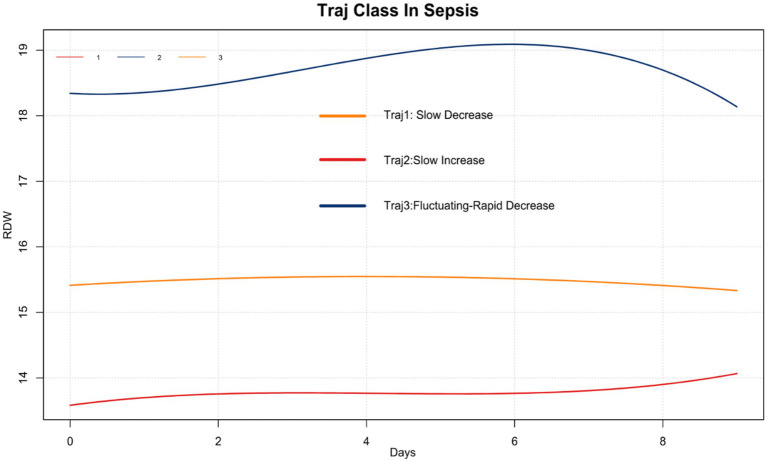
Validation of trajectories using data from the First Affiliated Hospital of Kunming Medical University.

We additionally collected clinical data from sepsis patients hospitalized at the First Affiliated Hospital of Kunming Medical University between 2018 and 2025. This dataset included detailed information such as basic demographics, diagnoses, outcomes, treatment regimens, and laboratory results, encompassing a total of 11,336 patients. After applying the same inclusion and exclusion criteria used for the modeling cohort, 467 patients were included in the validation study. We similarly employed GBTM to identify RDW trajectories in these patients. The resulting trajectory model closely resembled that derived from the MIMIC-IV database (as shown in Figure 5), also comprising three distinct trajectory groups: Trajectory 1 (Slow-Decrease group), Trajectory 2 (Slow-Increase group), and Trajectory 3 (Fluctuating-Rapid Decrease group).

**Figure 6:**
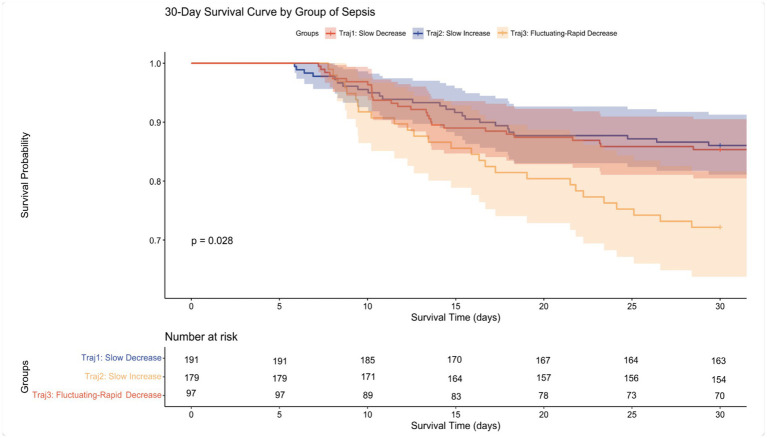
Kaplan–Meier survival curves for different trajectories derived from the First Affiliated Hospital of Kunming Medical University data.

After assigning patients to groups based on the maximum posterior probability, we plotted Kaplan–Meier survival curves for 30-day survival for the three groups. The results indicated that mortality in the Traj 3 group was substantially higher than in the other two groups, which was consistent with our model’s predictions. Pairwise Log-rank tests between the three different trajectories revealed that both the survival rate and survival time of Traj 3 patients were significantly different from those of Traj 1 and Traj 2 patients (*p* = 0.017 and *p* = 0.019, respectively), while no significant difference was observed between Traj 1 and Traj 2 (*p* = 0.793) (Figure 6).

## 4 Discussion

**Figure 7:**
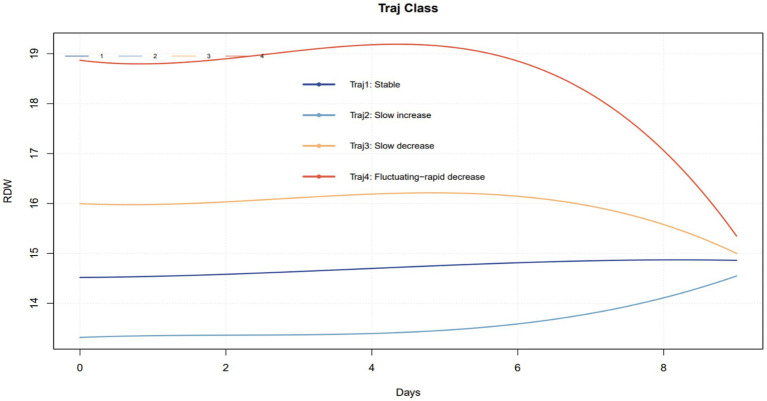
The trajectory plot of the four-trajectory model.

In this study, we compared models containing one to four potential trajectory groups based on the criteria of minimizing AIC and BIC. From the perspective of statistical fit, the four-trajectory model had the lowest AIC (99311.23) and BIC (99507.85) values (see Supplementary Table S3 for details), indicating a slightly better statistical fit to the current sample data than the three-trajectory model (AIC = 109842.06; BIC = 109987.39). However, model selection should not rely solely on statistical metrics but should be based more importantly on clinical interpretability and practical utility. We ultimately selected the three-trajectory model based on a comprehensive consideration of the following three aspects:

First, and most crucially, the newly added trajectory lacked independent clinical significance. As shown in Figure 7, compared to the three-trajectory model, the four-trajectory model primarily splits the ‘Slow Decrease’ group further into a ‘Stable’ group. However, subsequent survival analysis and multivariable Cox regression analysis demonstrated that this newly added ‘Stable’ group showed no statistically significant difference in 30-day and 90-day mortality compared to the original ‘Slow Decrease’ group (see Supplementary Table S3 for details). This indicates that, from the core clinical objective of prognostic stratification, distinguishing these two groups did not provide additional informational value. They essentially represent patient populations with similar risk profiles.

Second, adhering to the principle of model parsimony. The four-trajectory model increased model complexity without achieving meaningful improvement in the prediction of the core outcome. Therefore, the three-trajectory model achieves a better balance between simplicity and efficacy.

Finally, focusing on the feasibility of clinical translation. The ultimate goal of our research is to develop a dynamic risk assessment tool that is easy for clinicians to understand and apply. A model comprising three distinct trajectories with clear and prognostically different outcomes has a more intuitive classification logic, making it easier to grasp and utilize in busy clinical settings. Adding a fourth trajectory that is prognostically similar to an existing category yet potentially increases the complexity of judgment could diminish the practical value of the tool at the bedside. Consequently, after in-depth discussion within the research team and with clinical colleagues, we unanimously agreed that the three-trajectory model achieves the optimal integration of statistical adequacy, clinical interpretability, and practical simplicity, making it the superior choice for serving clinical decision-making.

Following the group-based trajectory modeling, we assigned each patient to one of the three different trajectory groups based on posterior probabilities. Using the Traj 1 group (Slow Decrease) as the reference, we performed Cox proportional hazards model and Kaplan–Meier survival curve analyses. We also validated the model’s reasonableness using local data from our hospital. In our established model, the Traj 3 group had the highest SOFA score and initial RDW value and was associated with the worst 30-day and 90-day prognosis. Conversely, the Traj 2 group had the longest ICU length of stay and the lowest initial RDW value and was associated with the best 30-day and 90-day prognosis, although its protective effect disappeared after multivariable adjustment. To our knowledge, this is the first study to dynamically analyze the trajectory of RDW changes during the course of sepsis.

RDW level is an indicator reflecting the variability in size of red blood cells in the circulation. Erythropoietin is the hormone that most significantly affects RDW. Abnormal production of erythropoietin and reduced responsiveness of red blood cells to erythropoietin can both lead to abnormally elevated RDW level (10), with the latter having a greater impact in sepsis. Inflammatory cytokines such as TNF-*α*, IL-1β, and IL-6 reduce the sensitivity of bone marrow erythroid cells to erythropoietin. This leads to the release of immature red blood cells into the bloodstream and increases ineffective erythropoiesis, thereby elevating RDW level. Studies have shown that the negative impact of sepsis on hematopoietic system function is reflected in the reduced lifespan of red blood cells during the hyperinflammatory state of sepsis (10), leading to increased red blood cell heterogeneity. This affects normal red blood cell production and contributes to increased RDW level (3, 10–13, 14). On the other hand, under inflammatory conditions, IL-6 also promotes the expression of hepcidin via the JAK-STAT3 pathway (15). Hepcidin is a core regulatory hormone of iron metabolism. It binds to the iron exporter ferroportin, leading to its degradation, thereby inhibiting iron efflux and causing impaired iron utilization. During inflammation, it blocks iron release from macrophages and intestinal iron absorption, leading to hypoferremia and anemia of inflammation, causing “anemia of inflammation.” This results in ineffective and heterogeneous red blood cell production, thereby elevating RDW (14). However, sepsis is often accompanied by hypoxia, which can directly inhibit hepcidin production via the HIF/transmembrane serine protease 6 (TMPRSS6)/membrane-bound serine protease Matriptase-2 (MT2) pathway (16), promoting iron release and accelerating red blood cell production, albeit with potentially reduced uniformity. In summary, changes in RDW during sepsis are the result of multiple factors; inflammation, impaired iron utilization, and hypoxia all influence RDW.

RDW not only has good diagnostic value in chronic inflammation but recent studies have also demonstrated its role in the diagnosis and prognosis prediction of acute inflammatory conditions like sepsis. For instance, studies by Eyiol et al. (17) and Eyiol et al. (18) found that in patients with acute myocarditis, a lower Hemoglobin to RDW Ratio (HRR) or a higher RDW to Albumin Ratio (RAR) was significantly associated with more severe clinical conditions such as pericardial effusion, need for inotropic support, or need for intravenous steroids or immunoglobulins. In patients with acute pericarditis, those presenting with pericardial effusion, ECG changes, pericardial friction rub, or requiring intravenous steroids had significantly lower HRR. Patients with the aforementioned symptoms requiring intravenous steroids or those with persistent chest pain at 15-day follow-up had significantly higher RAR (17, 18). Kim et al. found that sepsis patients with an increase in RDW within 72 h of Emergency Department admission had a higher risk of death (19). Wang et al. found that RDW level was an independent predictor of mortality in elderly ICU sepsis patients, with each 1% increase in RDW associated with an 18% increase in mortality (14). Gu et al. indicated that an elevated RDW to Albumin Ratio (RAR) was significantly associated with increased in-hospital mortality in sepsis patients with comorbid atrial fibrillation (20). Zeng et al. found that increased RDW was associated with in-hospital, short-term, and long-term mortality in critically ill patients with comorbid atrial fibrillation (21). Han et al. found that elevated RDW was associated with poor long-term prognosis in sepsis patients, and compared to five traditional severity scoring systems (APS III, MLODS, OASIS, SOFA, SIRS, SAPS II, and qSOFA), RDW had higher predictive accuracy (22). In another study by Kim et al., a prognostic prediction model for sepsis constructed using increased RDW (>14.5%), increased Delta Neutrophil Index (DNI) (>5.0%), or decreased platelet count (<150,000/mm^3^) demonstrated good predictive ability (AUC = 0.758) (23). Ku et al. found that the RDW level at the onset of bacteremia was an independent predictor of 28-day mortality (24). Kim et al. showed that an increase in RDW from baseline during the first 72 h of hospitalization could serve as a powerful independent predictor of mortality in patients with severe sepsis or septic shock (25). Notably, Fontana et al., by monitoring sublingual microcirculation, found that changes in RDW were not associated with microcirculatory alterations in sepsis patients (26). In our study, analysis after adding peripheral vascular disease as a confounder in the Cox model also showed it was not a risk factor for patient death, which aligns with the conclusion of Fontana et al.

Prior studies have demonstrated the predictive role of RDW as a relevant factor in sepsis. However, these studies focused solely on the value of RDW at a single time point, neglecting its dynamic changes. In the present study, we identified distinct RDW trajectories using group-based trajectory modeling and validated the model’s reasonableness and scientific validity with local data. The results indicate that dynamic RDW trajectories are not only significantly associated with patient prognosis but also provide prognostic information for sepsis patients that is independent of and incremental to traditional scoring systems. This is supported by consistent and strong evidence from the C-index and likelihood ratio tests. Multi-model validation showed that incorporating RDW trajectory grouping significantly improved the discrimination (C-index) and goodness-of-fit (all likelihood ratio test *p* < 0.001) of the prognostic models. NRI analysis further revealed that the improvement primarily stemmed from the optimization of risk classification for surviving patients, especially showing significant value in basic clinical models. Although its reclassification benefit was limited in the most comprehensive model, RDW trajectories still provided independent dynamic prognostic information. Furthermore, the Schoenfeld residual tests in the multivariate Cox proportional hazards models showed that the association strength between the RDW trajectory and mortality risk remained stable throughout the observation period (see Supplementary Tables S1, S2 for details). In contrast, some traditional static severity scores, such as the SOFA score, showed a trend of changing hazard ratios over time (*p* < 0.001). While this does not negate the clinical value of scores like SOFA, as their role in assessing organ dysfunction in sepsis patients at a fixed time point has been confirmed by numerous studies, our research suggests that the RDW trajectory, based on dynamic monitoring over the first 10 days, may provide a more stable risk stratification signal in the longitudinal dimension.

A limitation of this study is that to establish RDW trajectories for sepsis patients, we needed RDW values at different time points, thus requiring patients with multiple RDW measurements after admission. These patients often had more severe conditions necessitating frequent RDW monitoring, potentially excluding some patients with milder infections, a point that warrants attention. However, RDW is one of the most easily obtainable clinical parameters. It is closely related to the patient’s inflammatory status, and its testing cost is much lower compared to other inflammatory markers (such as cytokines). By dynamically monitoring a patient’s RDW trajectory and identifying the trajectory type, it may assist clinicians in early judgment of the patient’s condition and adjustment of treatment plans, ultimately improving patient prognosis and reducing the burden of sepsis hospitalization.

In summary, using GBTM, we identified three distinct RDW trajectories in sepsis patients. Traj 2 was associated with higher 30-day and 90-day survival rates, while its protective effect disappeared after multivariable adjustment. Traj 3 was associated with higher 30-day and 90-day mortality rates. This may be of assistance in the work of clinicians.

## Data Availability

Publicly available datasets were analyzed in this study. This data can be found at: https://physionet.org/content/mimiciv/.
