# Supplementary material for: Three distinct trajectories of red blood cell distribution width and their significant associations with mortality in sepsis patients: a group-based trajectory modeling study with validation
Source: Front Med (Lausanne). 2026 Apr 13;13:1816360. doi: 10.3389/fmed.2026.1816360 (PMC13111266; doi:10.3389/fmed.2026.1816360)
Supplement: Supplementary file 1 [file Data_Sheet_1.PDF]

# Supplementary Material

Table 1. Results of the Proportional Hazards Assumption Test (Schoenfeld Residual Test) for Variables in the 30-Day Survival Model

| Variable | Crude | Model I | Model II | Model III |
|---|---|---|---|---|
| Trajectories | 1.82 (2), 0.400 | 1.85 (2), 0.400 | 1.979 (2), 0.372 | 2.195 (2), 0.334 |
| Anchor age | | 1.06 (1), 0.300 | 1.031 (1), 0.310 | 1.065 (1), 0.302 |
| Gender | | 1.07 (1), 0.300 | 0.944 (1), 0.331 | 0.896 (1), 0.344 |
| ICU stay | | 24.36 (1), 8.0e-07 | 23.412 (1), 1.3e-06 | 27.123 (1), 1.9e-07 |
| Sofa | | | 10.287 (1), 0.001 | 11.189 (1), 0.001 |
| GCS | | | 4.227 (1), 0.040 | 3.663 (1), 0.056 |
| SIRS | | | 2.230 (1), 0.135 | 2.534 (1), 0.111 |
| APSIII | | | | 2.542 (1), 0.111 |
| OASIS | | | | 0.007 (1), 0.931 |
| Charlson | | | | 2.630 (1), 0.105 |
| **GLOBAL** | 1.82 (2), 0.400 | 28.03 (5), 3.6e-05 | 37.076 (8), 1.1e-05 | 48.988 (11), 9.5e-07 |

**Table Format:** $\chi^2$ (df), *p*-value, **Crude:** Unadjusted model.**Model I:** Adjusted for age, sex, and ICU length of stay.**Model II:** Adjusted for variables in Model I plus SOFA score, GCS score, and SIRS score.**Model III:** Adjusted for variables in Model II plus APS III score, OASIS score, and Charlson Comorbidity Index.

Table 2. Results of the Proportional Hazards Assumption Test (Schoenfeld Residual Test) for Variables in the 90-Day Survival Model

| Variable | Crude | Model I | Model II | Model III |
|---|---|---|---|---|
| Trajectories | 2.01 (2), 0.370 | 2.05 (2), 0.360 | 2.220 (2), 0.329 | 2.602 (2), 0.272 |
| Anchor age | | 16.54 (1), 4.8e-05 | 16.58 (1), 4.7e-05 | 17.271 (1), 3.2e-05 |
| Gender | | 1.62 (1), 0.200 | 1.45 (1), 0.228 | 1.475 (1), 0.224 |
| ICU stay | | 34.76 (1), 3.7e-09 | 33.25 (1), 8.1e-09 | 38.024 (1), 7.0e-10 |
| Sofa | | | 10.74 (1), 0.001 | 11.614 (1), 0.001 |
| GCS | | | 1.71 (1), 0.192 | 1.433 (1), 0.231 |
| SIRS | | | 3.25 (1), 0.071 | 3.567 (1), 0.059 |
| APSIII | | | 54.14 (8), 6.5e-09 | 4.931 (1), 0.026 |
| OASIS | | | | 3.088 (1), 0.079 |
| Charlson | | | | 0.326 (1), 0.568 |
| **GLOBAL** | 2.01 (2), 0.370 | 50.12 (5), 1.3e-09 | 54.14 (8), 6.5e-09 | 74.380 (11), 1.8e-11 |

**Crude:** Unadjusted model.**Model I:** Adjusted for age, sex, and ICU length of stay.**Model II:** Adjusted for variables in Model I plus SOFA score, GCS score, and SIRS score.**Model III:** Adjusted for variables in Model II plus APS III score, OASIS score, and Charlson Comorbidity Index.

Table 3. Univariate and Multivariate Cox Proportional Hazards Models for the Four-Group RDW Trajectory Model

| | Traj | Crude | Model I | Model II | Model III |
|---|---|---|---|---|---|
| 30-day mortality | Traj 1 | Ref | Ref | Ref | Ref |
| | Traj 2 | 0.61（0.46 0.80，*P*＜0.001） | 0.61（0.46, 0.80，*P*＜0.001） | 0.65（0.49, 0.85，*P*=0.002） | 0.73（0.55， 0.96，*P*=0.025） |
| | Traj 3 | 0.91（0.70, 1.18，*P*=0.472） | 0.92（0.71, 1.19，*P*=0.535） | 0.93（0.72, 1.21，*P*=0.594） | 0.82（0.63, 1.07，*P*=0.142） |
| | Traj 4 | 1.64（1.28, 2.09，*P*＜0.001） | 1.70（1.33, 2.18，*P*＜0.001） | 1.629（1.27, 2.09，*P*＜0.001） | 1.38（1.07, 1.78，*P*=0.012） |
| 90-day mortality | Traj 1 | Ref | Ref | Ref | Ref |
| | Traj 2 | 0.72（0.56, 0.92，*P*=0.009） | 0.70（0.55, 0.90，*P*=0.006） | 0.75（0.59, 0.97，*P*=0.028） | 0.86（0.67, 1.10，*P*=0.231） |
| | Traj 3 | 1.03（0.81, 1.30，*P*=0.821） | 1.05（0.83, 1.33，*P*=0.707） | 1.06（0.84, 1.35，*P*=0.622） | 0.94（0.74, 1.20，*P*=0.628） |
| | Traj 4 | 1.82（1.45, 2.28，*P*＜0.001） | 1.88（1.50, 2.36，*P*＜0.001） | 1.79（1.42, 2.25，*P*＜0.001） | 1.50（1.19, 1.90，*P*=0.001） |

**Crude:** Unadjusted model.**Model I:** Adjusted for age, sex, and ICU length of stay.**Model II:** Adjusted for variables in Model I plus SOFA score, GCS score, and SIRS score.**Model III:** Adjusted for variables in Model II plus APS III score, OASIS score, and Charlson Comorbidity Index.
